# Tubulin Polymerization Promoting Protein Affects the Circadian Timing System in C57Bl/6 Mice

**DOI:** 10.5334/jcr.207

**Published:** 2021-05-20

**Authors:** Eric Barbato, Rebecca Darrah, Thomas J. Kelley

**Affiliations:** 1 Department of Genetics and Genome Sciences, Case Western Reserve University, Cleveland Ohio, US

**Keywords:** circadian timing system, circadian rhythm, melatonin, microtubule, mouse, clock genes, locomotor activity

## Abstract

The circadian timing system (CTS) is a complex set of cyclic cellular mechanisms which serve to synchronize discrete cell groups across multiple organ systems to adapt the bodys physiology to a (roughly) 24-hour clock. Many genes and hormones have been shown to be strongly associated with the CTS, some of which include the genes *Bmal1, Period1, Period2, Cryptochrome1*, and *Cryptochrome2*, and the hormone melatonin. Previous data suggest that microtubule dynamics play an important role in melatonin function as it relates to the CTS in vitro, though this relationship has never been explored in vivo. The purpose of this study was to determine whether disruption of microtubule regulation in C57Bl/6 mice results in measurable changes to the CTS. To study the potential effects of microtubule dynamics on the CTS in vivo, we utilized a mouse model of microtubule instability, knocked out for the tubulin polymerization promoting protein gene (*Tppp -/-*), comparing them to their wild type (WT) littermates in three categories: locomotor activity (in light/dark and dark/dark photoperiods), serial clock gene expression, and serial serum melatonin concentration. These comparisons showed differences in all three categories, including significant differences in locomotor characteristics under dark/dark conditions. Our findings support and extend previous reports that microtubule dynamics are a modulator of circadian rhythm regulation likely through a mechanism involving melatonin induced phase shifting.

## Introduction

The circadian timing system (CTS) is a complex set of cyclic cellular mechanisms which serve to synchronize discrete cell groups across multiple organ systems to adapt the bodys physiology to a (roughly) 24-hour clock. Some of the physiologic functions regulated by the CTS include sleep, immune response, cardiac function, and many aspects of behavior [1,2,3]. The cell groups responsible for regulation of the CTS function as oscillators, in which multiple circadian clock genes are expressed at varying levels rhythmically throughout the day. That rhythmic expression is regulated by a self-contained feedback loop which is integral to the function of the CTS. Dysfunction of the system, caused by endogenous or exogenous factors, has been linked to many disease processes, including disordered sleep, anxiety and depression, cardiac disease, immune dysfunction, and many others [4,5].

Many genes and their protein products have been associated with the CTS, some of which include *Clock, Bmal1, Period1, Period2, Cryptochrome1*, and *Cryptochrome2* [5,6]. The core molecular functionality of the CTS, including its unique process of self-regulating gene expression, has been well described [4,7,8]. Additionally, the manner by which certain exogenous factors such as light or sound can entrain or affect the system has been characterized [9]. There are also many endogenous factors known to entrain the CTS to some degree, including expression of certain genes and proteins, as well as melatonin production.

Melatonin, a hormone secreted by the mammalian pineal gland, is a powerful molecular regulator of sleep/wake cycles in diurnal species [10]. In humans and mice, melatonin secretion in the pineal gland is driven by the suprachiasmatic nucleus (SCN) of the hypothalamus as a response to external light input via non-rod, non-cone photoreceptors [11,12,13]. Melatonin binds to the G-protein coupled receptors MT_1_ and MT_2_ in the SCN and peripheral tissues, thereby effecting physiologic changes in those systems [14]. In this way, these species are able to effectively adapt their biological rhythms to changes in environmental light, such as the daily light/dark (LD) cycle or the change of light and dark period durations during the seasons.

MT_1_ and MT_2_ receptor function and consequent melatonin-related CTS changes can be affected by many factors [15]. Previous data suggest that microtubule dynamics are a powerful modulator of melatonin receptor function and sensitivity in both rodent and human cells [16,17,18]. Although the mechanisms underlying this interaction have been studied in vitro, potential phenotypic changes to the circadian system resulting from microtubule manipulation have yet to be investigated in vivo.

The hypothesis of this study is that direct manipulation of microtubule stability will disrupt normal circadian regulation. To study the potential phenotypic effects of microtubule dynamics on the CTS in vivo, we utilized a mouse model of microtubule instability, knocked out for the tubulin polymerization promoting protein gene (*Tppp -/-*). Its protein product, TPPP, serves as an essential component of microtubule dynamics and stability, promoting microtubule polymerization and regulating tubulin acetylation through the inhibition of histone deacetylase 6 (HDAC6) activity [19]. Altered regulation of TPPP has been associated with phenotypes in Parkinsons disease and Alzheimers disease [20,21,22]. We have demonstrated a role for microtubule dysregulation in several phenotypes in cystic fibrosis (CF), phenotypes that can be mimicked in WT cells by knock-down of TPPP expression and reversed by inhibition of HDAC6 [23,24,25,26]. Knock-down of TPPP expression in WT cells resulted in slowed microtubule formation rates and reduced tubulin acetylation [23]. Each of these diseases is characterized in part by disordered sleep patterns [27,28,29,30,31,32]. In this study it is determined that *Tppp* null mice exhibit reduced melatonin expression, altered circadian rhythm related gene expression profiles, reduced amplitude activity rhythms, and phase shifted sleep/activity cycles compared to WT controls. In addition, *Tppp* null mice showed altered activity characteristics under dark:dark (DD) conditions, suggesting a role for TPPP in endogenous timekeeping. Thus, it is concluded that TPPP is a contributor to the regulation of CTS, likely through the alteration of microtubule stability.

## Materials and methods

### Categories for evaluation

To test our hypothesis, we compared *Tppp -/-* mice to their wildtype (WT) littermates in three key categories. The first category was locomotor activity, which was measured continuously in identical environments for five days under 12:12 LD conditions and 21 days under DD conditions. The second category was clock gene expression, which included serial quantification of *Clock, Bmal1, Period1, Period2, Cryptochrome1*, and *Cryptochrome2* mRNA isolated from dissected suprachiasmatic nucleus (SCN) tissue. The third and final category was serial serum melatonin concentration. Both clock gene expression and serum melatonin were measured at four time points throughout the LD photoperiod following completion of the 12:12 LD locomotor activity data collection.

### Animals and measurement of locomotor activity

The experimental protocols described in this report including animal breeding, housing, surgery, behavioral testing, sacrifice, and tissue collection, were approved by the Institutional Animal Care and Use Committee of Case Western Reserve University, Cleveland, Ohio. C57Bl/6 mice of two genotype groups were utilized for these experiments: WT and *Tppp -/-*, which were homozygous for a null mutation in the coding region of *Tppp*. Both groups consisted of 12 mice each; six males and six females. Mice in both groups ranged from 713 weeks old, averaging 9.6 weeks at the start of the data collection period. Each mouse was implanted with a subcutaneous telemetry device and allowed a four-day recovery period before acclimation to a light-tight circadian cabinet in which the mice were housed for the duration of the experiments with access to food and water ad-libitum.

Implantation surgery was performed in sterile conditions under anesthesia with nebulized isoflurane. Once a surgical plane of anesthesia was confirmed by toe pinch, each mouse was pre-treated with analgesia, secured to the heated surgical table, and a ~2sq cm area was shaved on the dorsal fur between the base of the skull and scapulae. Following preparation of the area with betadine, a ~1 cm superficial incision was made and gently spread posteriorly with surgical scissors. Using forceps, a G2 E-Mitter telemetry device (Starr Life Sciences) which had previously been sterilized and rinsed in sterile saline, was implanted in the incision site. The surgical incision was then closed using a single wound clip and the mouse was placed into a standard mouse cage atop a heating pad. Post-surgical analgesia was administered as needed for the following three days of recovery.

After the four-day recovery period, mice were placed one per cage atop an ER-4000 Energizer/Receiver (Starr Life Sciences), a receiving plate slightly larger than the standard mouse cage designed to receive telemetry data from the E-Mitter device. Each cage/receiver combination was placed in a custom circadian cabinet (Actimetrics) which is designed to house eight mouse cages simultaneously and is shielded from exterior light, temperature, and humidity. The mice were then allowed to acclimate to the cabinet environment for one week at constant temperature (22C 3C) on a constant 12:12 LD photoperiod identical in timing to the lights of the room in the animal facility in which they had been previously housed. Following the acclimation period, the E-Mitter/ER-4000 equipment was activated and continuous gross motor activity data were collected.

Due to the photoperiod timing restrictions of our experimental design, we used separate mice for the 12:12 LD and DD experiments. Eight mice of each genotype were analyzed under 12:12 LD photoperiod for five consecutive days. During the dark phase of the sixth day, mice were taken from the cabinet in groups of two (one of each genotype) for euthanasia at three time points: ZT 6, ZT 12.5, ZT 18, and ZT 23.5. Since the circadian cabinet is capable of handling only eight cages at once, we repeated these experiments under identical conditions once to increase sample sizes of each group. For the DD experiments, eight mice (four of each genotype) were implanted, allowed to recover, and placed in the cabinet with the lights off. The mice were allowed a two-week acclimation period before the 22-day data collection period.

**Figure 1:**
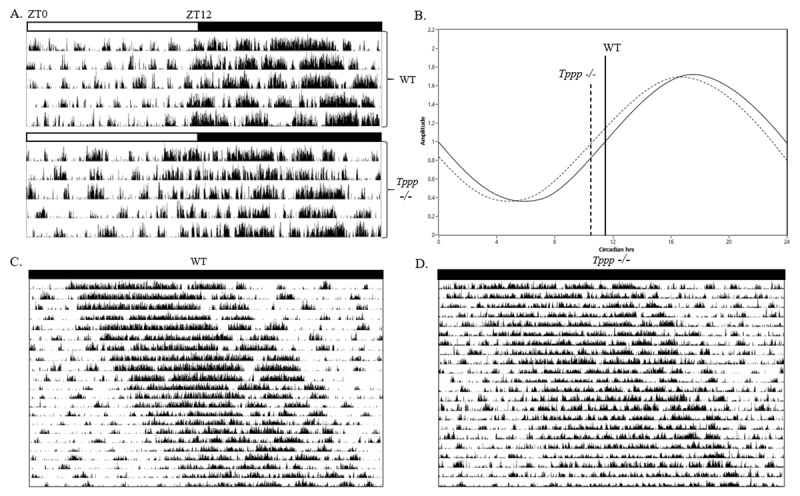
**(A)** Representative actograms of *Tppp -/-* (top) and WT (bottom) during 12/12 LD photoperiod. Actogram data are single plotted (24h per line) with one line representing each day of the LD experiment. **(B)** Sin fit waves representing locomotor activity profiles of WT (solid line, n = 8) and *Tppp -/-* mice (dashed line, n = 8) with Circadian hours on the x-axis and signal amplitude on the y-axis. A solid vertical line (WT) and dashed vertical line (*Tppp -/-*) represent mean activity onset time of each group. **(C & D)** Representative actograms of WT (C) and *Tppp -/-* **(D)** and during DD photoperiod.

Locomotor activity data were analyzed using Clocklabs Analysis 6.0 (Actimetrics). Single-plotted actograms and activity profiles were also generated using Clocklabs Analysis (***Figure 1***). Independent samples t-tests were conducted to compare relevant phenotypic characteristics of the *Tppp -/-* group to the WT group for which statistical significance was considered p = 0.05 (***Tables 1*** and ***2***).

**Table 1:** Phenotypic characteristics of WT (n = 8) and *Tppp -/-* (n = 8) mice during 12/12 LD photoperiod. Values are displayed as mean SEM apart from rows representing FFT values, which are displayed as variance.


LD CHARACTERISTIC	WT	*TPPP -/-*	T-TEST P VALUE

LD Period (hr.)	23.98 .11	23.98 .12	.34

Mean LD activity/hour (counts)	32.17 3.6	26.89 2.5	<.001

Mean acrophase (hr.)	17.69 .12	16.73 .18	.01

Mean trough (hr.)	5.7 .11	4.73 .15	.03

LD activity onset relative to lights off (hr.)	.01 .10	.89 .11	.005

FFT circadian amplitude (% total variance)	.38 .14	.37 .16	.55

FFT ultradian amplitude (% total variance)	.08 .02	.04 .02	.09


**Table 2:** Phenotypic characteristics of WT (n = 4) and *Tppp -/-* (n = 4) mice during DD photoperiod. Values are displayed as mean SEM for DD period and mean activity, mean variance for FFT values.


DD CHARACTERISTIC	WT	*TPPP -/-*	T-TEST P VALUE

DD Period (hr.)	23.78 .08	24.63 .19	.002

Mean DD activity/hour (counts)	30.21 2.3	26.89 1.5	.001

FFT circadian amplitude (% total variance)	.31 .1	.25 .16	.04

FFT ultradian amplitude (% total variance)	.06 .01	.01 .01	.004


### Tissue collection, RNA extraction, and gene expression analyses

On the day of euthanasia, animals were taken from the circadian cabinet in pairs (one of each genotype) and euthanized at ZT 6, ZT 12.5, ZT 18, and ZT 23.5. Mice were euthanized via CO2 exposure and SCN was immediately dissected and flash frozen in liquid nitrogen. Each frozen tissue sample was then stored at 80C for later use. Total RNA was extracted from each sample using the RNeasy Mini Kit (Qiagen) according to manufacturers specifications. RNA quality and concentration were quantified using a Nanodrop spectrophotometer (ThermoFisher) and 1g of total RNA was converted to cDNA using the qScript cDNA Synthesis Kit (Quantabio) according the manufacturers protocol.

To determine relative expression of each gene transcript, we performed quantitative real-time PCR using standard TaqMan assays (ThermoFisher) with -actin as an endogenous control. mRNA transcripts evaluated for expression were: *Clock* (Mm00455950_m1), *Bmal1* (Mm00500226_m1), *Period1* (Mm00501813_m1), *Period2* (Mm00478113_m1), *Cryptochrome1* (Mm00514392_m1), and *Cryptochrome2* (Mm01331539_m1). Each reaction was denatured at 95C and amplified at 60C for 50 cycles alongside cDNA controls with no reverse-transcriptase to verify the absence of detectable genomic carryover in the RNA isolates. Cycle thresholds (Ct) were calculated using StepOnePlus software and mean Ct difference (Ct) was calculated between each gene of interest and endogenous controls. Genotype groups (*Tppp -/-* and WT) were then compared by calculating Ct difference between each for each transcript (Ct). Fold difference of the *Tppp -/-* group (2^Ct^) is reported as a percent of the WT control group.

**Figure 2:**
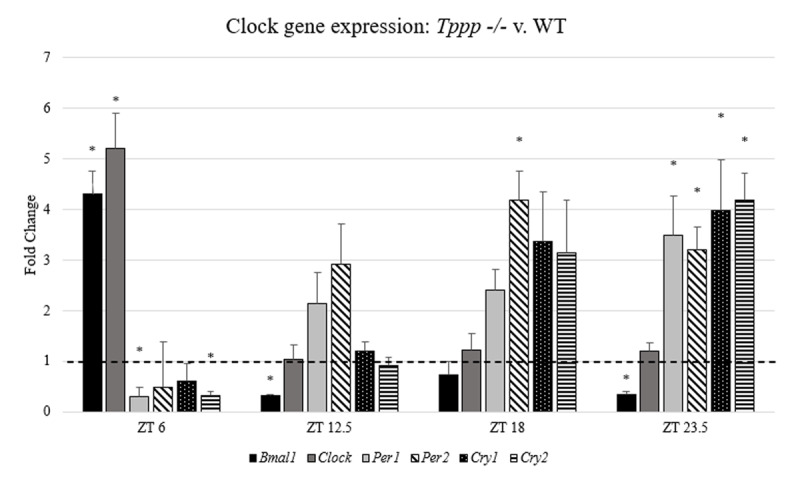
Serial clock gene expression in SCN; *Tppp -/-* v. WT at three time points. Values are displayed as *Tppp -/-* fold change relative to WT (the WT value for each transcript is represented by the dashed line at 1.00 on the y-axis). Asterisks indicate statistical significance at p < 0.008. Error bars represent SEM.

Independent samples t-tests were conducted to compare average fold difference between the *Tppp -/-* group and the WT group for each transcript at each time point. After conducting the Bonferroni correction for multiple comparisons, statistical significance for these comparisons was considered p = 0.008 (***Figure 2***).

### Serum extraction and melatonin measurement

It has been reported that rodents produce very small concentrations of melatonin which are often difficult to detect [33]. Consequently, to accurately evaluate circulating melatonin concentration at multiple time points, it was necessary to use as much serum as possible for each sample, prohibiting a survival extraction technique such as orbital or tail vein blood draw. Immediately following euthanasia and alongside tissue dissection, a cardiac puncture was performed and whole blood was collected via sterile bulb pipette. Whole blood was placed in sterile 1.5 ml serum separator tubes (BD) and spun at 10,000 g for 7 minutes at room temperature to separate serum from other blood components. Serum was then extracted, lyophilized using a SpeedVac at room temperature, and frozen at 20C for later use. Lyophilized serum was reconstituted and melatonin concentration was measured according to manufacturers protocol using a Melatonin ELISA Kit (ENZ-KIT150-0001) (Enzo Life Sciences).

Independent samples t-tests were conducted to compare mean serum melatonin concentration between the *Tppp -/-* group and the WT group for at each time point. After conducting the Bonferroni correction for multiple comparisons, statistical significance for these comparisons was considered p = 0.01. In addition, change in mean melatonin concentration of each group across the four measured timepoints was evaluated using repeated-measures ANOVA.

## Results

### Locomotor activity

WT and *Tppp -/-* mice showed numerous differences in locomotor activity characteristics throughout the 12:12 LD photoperiod including onset time of activity rhythms in both the circadian and ultradian ranges as measured by Fast Fourier Transform (FFT) and mean counts per hour throughout the LD photoperiod. Descriptive statistics of locomotor activity, as well as circadian and ultradian periodicity in both groups can be found in ***Table 1***.

No differences were detected in LD period between the *Tppp -/-* group and WT. High amplitude signal in the circadian range and low amplitude signal in the ultradian range were detected with no significant differences between the WT and *Tppp -/-* groups. The *Tppp -/-* group, however, showed a significantly earlier mean timing of activity onset relative to lights off time (.89 hrs) than the WT group (+.01 hrs). Additionally, the mean acrophase and trough timing of the Tppp -/- group (16.73 hrs, 4.73 hrs) was similarly earlier than their WT counterparts (17.69 hrs, 5.7 hrs). Mean activity per hour measured in counts was also significantly lower during the LD photoperiod in the *Tppp -/-* group (26.89) compared to WT (32.17). A visual representation of these differences in locomotor activity patterns can be seen in ***Figure 1***.

In addition to the differences we observed during the 12:12 LD photoperiod, we also noted key locomotor activity characteristic differences between *Tppp -/-* and WT mice during the DD photoperiod including onset time of activity rhythms in the circadian and ultradian ranges as measured by Fast Fourier Transform (FFT) and mean counts per hour. Descriptive statistics of both groups during the DD photoperiod can be found in ***Table 2***.

A significant difference was detected in DD period between the *Tppp -/-* group (23.78 hrs) and WT (24.63 hrs), *t*(3) = 7.71, p = 0.002. In addition, FFT analysis revealed high amplitude circadian signal and low amplitude ultradian signal in both groups with the *Tppp -/-* group significantly lower compared to WT in both measurements (*t_circadian_*(3) = 2.45, p = 0.04) (*t_ultradian_*(3) = 6.12, p = 0.004). Notably, the low amplitude ultradian signal in the *Tppp -/-* group was nearly undetectable during the DD photoperiod. Additionally, mean activity per hour measured in counts was significantly lower during the in the *Tppp -/-* group (26.89) compared to WT (32.17), *t*(3) = 10.18, p = 0.001. A visual representation of these differences in locomotor activity patterns can be seen in ***Figure 1***.

### Gene expression

Significant differences were detected in the expression of all six measured transcripts at one or more timepoints in the *Tppp -/-* group compared to WT (***Figure 2***).

At ZT 6 (during the light photoperiod), *Bmal1* and *Clock* were significantly overexpressed in the *Tppp -/-* group compared to WT (*t_Bmal1_*(14) = 4.75, p < 0.001) (*t_Clock_*(14) = 5.31, p < 0.001). *Per1* and *Cry2* were significantly downregulated in the *Tppp -/-* group (*t_Per1_*(14) = 2.96, p = 0.005) (*t_Cry2_*(14) = 3.98, p < 0.001). *Per2* and *Cry1* showed a downward trend, but were not significant at the p = 0.008 level.

At all three dark photoperiod timepoints, expression of *Bmal1* trended down and was significantly downregulated at ZT 12.5 (*t*(14) = 2.88, p = 0.005) and ZT 23.5 (*t*(14) = 3.16, p = 0.003). *Per1* and *Per2* trended up at all three timepoints, showing significant upregulation in *Per2* at ZT 18 (*t*(14) = 4.67, p < 0.001) and both *Per1* and *Per2* at ZT 23.5 (*t_Per1_*(14) = 2.96, p = 0.005) (*t_Per2_*(14) = 3.98, p < 0.001). Expression of *Cry1* and *Cry2* were unchanged at ZT 12.4, trended up at ZT 18, and were significantly upregulated at ZT 23.5 (*t_Cry1_*(14) = 2.80, p = 0.006) (*t_Cry2_*(14) = 5.74, p < 0.001).

### Melatonin concentration

**Figure 3:**
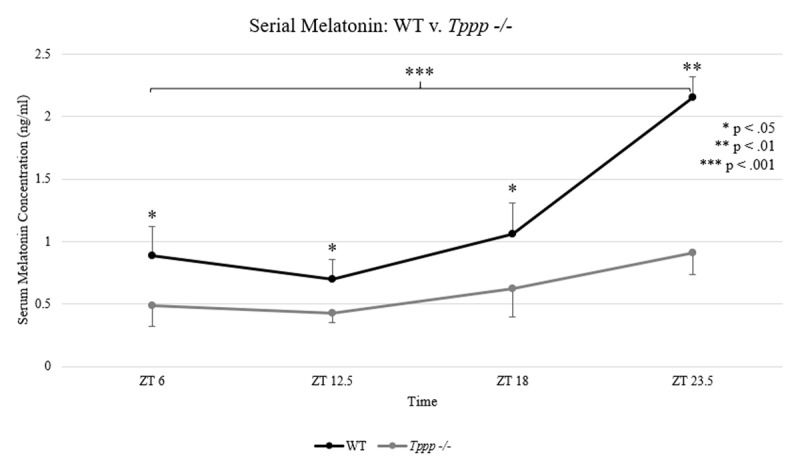
Serum melatonin concentration; WT (black) v. *Tppp -/-* (gray) at four timepoints (n = 3 per group per timepoint). Mean concentrations are displayed in ng/ml of serum. Asterisks indicate statistical significance for comparisons between groups (above each time point) and within the WT group over time (shown by the bracket).

Significant differences were detected in serum melatonin concentration between the *Tppp -/-* and WT groups at all four timepoints. Additionally, the rate at which serum melatonin concentration increased across the four measurement points was dampened in the *Tppp -/-* group compared to the control group (***Figure 3***).

At the first timepoint (ZT 6), mean melatonin concentration was significantly lower in the Tppp -/- group (0.49 ng/ml) compared to WT control (0.89 ng/ml), *t*(3) = 2.22, p = 0.03. At the second timepoint (ZT 12.5), mean serum melatonin concentration in *the Tppp -/-* group (0.43 ng/ml) was significantly lower than the WT control group (0.7 ng/ml), *t*(4) = 2.18, p = 0.04. At the third timepoint (ZT 18), mean of the *Tppp -/-* group (0.63 ng/ml) was again lower than WT (1.06 ng/ml), *t*(3) = 2.28, p = 0.04. At the fourth timepoint (ZT 23.5), mean of the *Tppp -/-* group (0.91 ng/ml) was again low compared to WT (2.15 ng/ml) showing the largest difference in group means at this measurement, *t*(4) = 7.23, p < 0.001.

In addition to lower mean concentrations at each individual timepoint, we detected a statistically significant effect of time on mean melatonin concentrations in the *Tppp -/-* group compared to WT controls. Mean serum melatonin in the WT group showed a statistically significant increase over time, *F*(1, 3) = 33.5, p = 0.003. Conversely, the *Tppp -/-* group did not show a significant change in mean melatonin concentration over the measurement period, *F*(1, 3) = 4.55, p = .09.

## Discussion

This study demonstrates that loss of expression of *Tppp* in C57Bl/6 mice results in a decrease in melatonin production accompanied by a phase advance in locomotor activity and clock gene expression. Given the patterns we observed in serum melatonin concentration and clock gene expression in the SCN in our experimental and control groups accompanied by phenotypic differences in both LD and DD photoperiods, we conclude that *Tppp* serves as a regulator of the CTS concurrent with modulation of melatonin production.

We observed no differences between WT and *Tppp -/-* mice in LD periodicity or length of time between mean trough and mean acrophase. Additionally, FFT data show no significant amplitude differences between groups in the circadian or ultradian ranges during the LD photoperiod. However, both mean trough and mean acrophase of the *Tppp -/-* group were shifted almost exactly one hour earlier than WT controls during that light schedule. Given that all mice were kept in identical conditions for the LD experiments, these data indicate that *Tppp -/-* mice retain some ability to entrain their CTS in response to a 24h light schedule, though they exhibit a phase advance of one hour.

Given the differences we observed in locomotor activity characteristics during the LD photoperiod, it was necessary to repeat the experiment in DD conditions to determine whether loss of function of TPPP is associated with endogenous CTS control. Indeed, the DD period of the *Tppp -/-* group was significantly different under these conditions, indicating that TPPP plays some role in endogenous timekeeping. In addition, several other phenotypic differences were observed. Comparison of FFT analysis between groups in the DD photoperiod revealed significant amplitude differences in both the circadian and ultradian ranges. Additionally, comparison of FFT analysis between LD and DD photoperiods revealed that under DD conditions, both circadian and ultradian amplitudes were decreased in both groups. Perhaps our most notable FFT finding, however, was that under DD conditions, the ultradian signal of the *Tppp -/-* group was so low as to be nearly undetectable. Taken together, these results indicate that microtubule instability resulting from loss of function of TPPP plays a role in the keeping of endogenous rhythmicity.

The locomotor activity phase advance we observed during the LD photoperiod also seems to be present in the clock gene expression profile of the *Tppp -/-* group compared to WT. Here, we report significant differences in expression of *Bmal1, Per1, Per2, Cry1*, and *Cry2* at one or more timepoints. At ZT 12.5, though *Per1* and *Per2* trended up, the only statistically significant difference between groups was *Bmal1*, which was severely downregulated. At ZT 18, *Per1, Cry1* and *Cry2* trended up, but the only significant difference was a severe upregulation in *Per2*. At ZT 23.5, *Bmal1* was again downregulated but every other transcript with the exception of *Clock* was significantly upregulated.

It has been previously reported that *Clock* and *Bmal1* expression generally covaries and cycles in roughly 24 hours [34,35,36]. Considering these data, our finding that *Clock* remained unchanged in the *Tppp -/-* group even when *Bmal1* was severely downregulated is notable. However, it has also been reported that melatonin increases expression of *Bmal1* [37]. Given that we saw marked decreases in circulating melatonin in the *Tppp -/-* group compared to WT, our observation of decreased *Bmal1* expression in that group may be an effect of decreased melatonin.

Similar to *Clock* and *Bmal1*, it is known that expression of *Per* and *Cry* generally varies together again cycling in a roughly 24-hour timeframe [34,35,36]. Given that the PER:CRY heterodimer serves as repressive transcription factor for *Clock* and *Bmal1*, it is perhaps not surprising that we generally saw decreases in expression of *Bmal1* when *Per* and *Cry* were upregulated. Coupled with our other findings, including the phase advance in locomotor activity, it is our contention that the *Tppp -/-* group displayed a clock gene expression profile that was also advanced in phase.

According to previous reports, the rhythmic expression of *Bmal1* reaches its peak around ZT 12 in a 12:12 LD photoperiod [34,35,36]. Conversely, *Per* and *Cry* reach their troughs in transcriptional activity around the same time and are thus typically inversely proportional to *Bmal1*. Using these normal oscillations as a template, our clock gene expression findings in the *Tppp -/-* group may reflect a phase advance relative to the WT group similar to what we observed in locomotor activity. At the first timepoint, *Bmal1* in the *Tppp -/-* group may have already reached its peak in transcriptional activity and started to downregulate, explaining its reduced expression during this and subsequent measurements, all of which were during the active phase when *Bmal1* transcription normally decreases. Similarly, at the first timepoint, *Per* and *Cry* may have already reached their trough in expression in the *Tppp -/-* group and started to upregulate. This, again, would explain the relative increases in *Per* and *Cry* throughout the measurement period when expression of those genes is normally increasing.

Finally, we observed that the *Tppp -/-* group showed significantly decreased serum melatonin concentration compared to WT at all three timepoints. Also of note, the rate at which concentration increased across the three measurement points was significantly reduced in the *Tppp -/-* group. These findings may help to explain the phase advances described above. As previously stated, it has been reported that melatonin drives transcription of *Bmal1* [37]. Furthermore, the crucial role of *Bmal1* as a pacemaker for the rhythmic expression of the other clock genes has been well described [38]. Coupled with our findings, these data indicate that decreased expression of *Bmal1* in the *Tppp -/-* group, perhaps as a result of decreased melatonin concentration, may be a causative factor in the striking phase advances we observed.

While further investigation is needed to determine the specific mechanisms underlying the phase advances we report here, our findings indicate clear melatonin deficiency and altered circadian rhythm regulation at multiple levels in a mouse model of microtubule dysfunction. These observations have clinical relevance in that microtubule dysfunction is a feature common to many diseases associated with CTS disturbances, including (but not limited to) cystic fibrosis, Rett syndrome, and Charcot-Marie-Tooth disease [39,40,41]. Subsequent research on this topic might apply the parameters measured here to these and other diseases associated microtubule dysfunction. In support of this hypothesis, the findings of this study are consistent with a recent manuscript examining a mouse model of schizophrenia where the expression of the microtubule binding and stabilizing protein, stable tubulin only protein (STOP), is knocked-out [42,43]. In two studies, the STOP knock-out mice exhibit fragmented sleep, less time asleep under light/dark cycle and constant dark conditions, and reduced REM sleep [42,43]. The authors concluded that the absence of the microtubule stabilizing protein STOP in mice resulted in clearly altered sleep physiology and activity cycles [42,43]. Together with the data presented here, two proteins that directly influence microtubule stability are shown to regulate aspects of the CTS.

In summary, our findings support previous reports that microtubule dynamics are a modulator of circadian rhythm regulation potentially through a mechanism involving melatonin induced phase shifting [16,17,18]. Additionally, our findings may highlight a common mechanism that influences sleep control in a number of clinical conditions and could point to the development of a common therapeutic intervention.
